# Serum brain‐derived neurotrophic factor and progression to MCI in cognitively normal older adults

**DOI:** 10.1002/alz.088177

**Published:** 2025-01-09

**Authors:** Kyungtae Kim, Min Soo Byun, Dahyun Yi, Joon Hyung Jung, Bo Kyung Sohn, Gijung Jung, Hyejin Ahn, Jun‐Young Lee, Yun‐Sang Lee, Yu Kyeong Kim, Dong Young Lee

**Affiliations:** ^1^ Department of Neuropsychiatry, Seoul National University Hospital, Seoul Korea, Republic of (South); ^2^ Department of Psychiatry, Seoul National University College of Medicine, Seoul Korea, Republic of (South); ^3^ Institute of Human Behavioral Medicine, Medical Research Center, Seoul National University, Seoul Korea, Republic of (South); ^4^ Department of Psychiatry, Chungbuk National University Hospital, Cheongju Korea, Republic of (South); ^5^ Department of Psychiatry, Inje University Sanggye Paik Hospital, Seoul Korea, Republic of (South); ^6^ Interdisciplinary program of cognitive science, Seoul National University, Seoul Korea, Republic of (South); ^7^ Department of Neuropsychiatry, SMG‐SNU Boramae Medical Center, Seoul Korea, Republic of (South); ^8^ Department of Nuclear Medicine, Seoul National University College of Medicine, Seoul Korea, Republic of (South); ^9^ Department of Nuclear Medicine, SMG‐SNU Boramae Medical Center, Seoul Korea, Republic of (South); ^10^ Interdisciplinary program of cognitive science, Seoul National University College of Humanities, Seoul Korea, Republic of (South)

## Abstract

**Background:**

Brain derived neurotropic factor (BDNF) has been associated with improved neuronal survival and synaptic plasticity and long‐term memory. Some human studies also reported the relationship between lower blood BDNF levels and poorer memory function and dementia. A prior longitudinal study also demonstrated higher serum BDNF levels were associated with lower risk of overall dementia and Alzheimer’s disease (AD) dementia. However, it remains uncertain whether reduced serum BDNF levels precede the onset of mild cognitive impairment (MCI), which is known as pre‐dementia stage. This study aimed to examine whether higher serum BDNF in cognitively normal (CN) older adults is related to less common progression to MCI and to identify potential moderators of this relationship.

**Method:**

A total of 278 CN older adults between 55 and 90 years of age were enrolled. All participants underwent comprehensive clinical assessments, serum BDNF level measurement, and multimodal brain imaging including Pittsburgh compound B (PIB)‐positron emission tomography (PET) at baseline, and followed up for up to 4 years. Serum BDNF levels were divided into two categories by median value of serum BDNF level: < 21448.5 pg/mL (low BDNF), and > 21448.5 pg/mL (high BDNF). Cox model was used to analyze the relationship between baseline BDNF levels and the risk for MCI progression controlling for potential confounders. We also ran sensitivity analyses stratified by sex, age, education, apolipoprotein E ε4 (APOE4) positivity, and amyloid PET positivity.

**Result:**

During follow‐up, 24 participants developed MCI. Controlling for age, sex, education and APOE4 positivity, low BDNF group had significantly more frequent progression to MCI than high BDNF group (hazard ratio for MCI, 2.98; 95% CI, 1.17‐7.56; p=0.02) and these association persisted even after controlling for amyloid PET positivity and vascular risk factor score as additional covariates (hazard ratio, 2.98; 95% CI, 1.17‐7.55; p=0.02). Sensitivity analyses revealed that the associations were apparent only among women, participants aged younger than 75 years, those without college degrees, and amyloid negative participants (Table).

**Conclusion:**

Higher serum BDNF levels may protect against future occurrence of MCI in cognitively healthy older adults. Such influence appears more prominent in women and younger, less educated, or amyloid PET negative individuals.

